# Maternal Diabetes Leads to Adaptation in Embryonic Amino Acid Metabolism during Early Pregnancy

**DOI:** 10.1371/journal.pone.0127465

**Published:** 2015-05-28

**Authors:** Jacqueline Gürke, Frank Hirche, René Thieme, Elisa Haucke, Maria Schindler, Gabriele I. Stangl, Bernd Fischer, Anne Navarrete Santos

**Affiliations:** 1 Department of Anatomy and Cell Biology, Martin Luther University Faculty of Medicine, Halle (Saale), Germany; 2 Department of Agricultural and Nutritional Sciences, Martin Luther University Halle-Wittenberg, Halle (Saale), Germany; Pennington Biomedical Research Center/LSU, UNITED STATES

## Abstract

During pregnancy an adequate amino acid supply is essential for embryo development and fetal growth. We have studied amino acid composition and branched chain amino acid (BCAA) metabolism at day 6 p.c. in diabetic rabbits and blastocysts. In the plasma of diabetic rabbits the concentrations of 12 amino acids were altered in comparison to the controls. Notably, the concentrations of the BCAA leucine, isoleucine and valine were approximately three-fold higher in diabetic rabbits than in the control. In the cavity fluid of blastocysts from diabetic rabbits BCAA concentrations were twice as high as those from controls, indicating a close link between maternal diabetes and embryonic BCAA metabolism. The expression of BCAA oxidizing enzymes and BCAA transporter was analysed in maternal tissues and in blastocysts. The RNA amounts of three oxidizing enzymes, i.e. branched chain aminotransferase 2 (Bcat2), branched chain ketoacid dehydrogenase (Bckdha) and dehydrolipoyl dehydrogenase (Dld), were markedly increased in maternal adipose tissue and decreased in liver and skeletal muscle of diabetic rabbits than in those of controls. Blastocysts of diabetic rabbits revealed a higher Bcat2 mRNA and protein abundance in comparison to control blastocysts. The expression of BCAA transporter LAT1 and LAT2 were unaltered in endometrium of diabetic and healthy rabbits, whereas LAT2 transcripts were increased in blastocysts of diabetic rabbits. In correlation to high embryonic BCAA levels the phosphorylation amount of the nutrient sensor mammalian target of rapamycin (mTOR) was enhanced in blastocysts caused by maternal diabetes. These results demonstrate a direct impact of maternal diabetes on BCAA concentrations and degradation in mammalian blastocysts with influence on embryonic mTOR signalling.

## Introduction

Amino acids are essential nutrients for embryo and fetal development and growth [1]. During pregnancy the availability and concentration of amino acids are vital determinants for trophoblast differentiation and implantation [2,3]. In vitro studies showed the necessity of amino acids for the development of zygotes in vitro (sheep) [4] and for preimplantation embryos from the golden hamster [5], bovine [6], mouse [7] and human [8]. In 1966, Gwatkin reported that an absence of the amino acids leucine and arginine in culture medium led to inhibited trophoblast growth [9].

The branched chain amino acids (BCAA) leucine, isoleucine and valine are of great importance for fetal growth. They are preferentially transported to the embryo via system L [10] and spread quickly across the placenta [11]. BCAA are three of the nine essential amino acids and are abundant in foods, accounting for 15 to 20% of total protein intake [12]. In contrast to other amino acids, BCAA are poorly metabolised in the liver. Sixty percent of them are metabolised in skeletal muscle [13]. Skeletal muscle and adipose tissue have the highest BCAA degradation capacity [14]. The BCAA metabolism encompasses three steps: transamination, oxidative decarboxylation and dehydration. The mitochondrial branched chain aminotransferase (BCAT2 or Bcatm) catalyses the transamination reaction as first step. BCAT2 is expressed in most peripheral tissues [15,16]. The branched chain ketoacid dehydrogenase complex (BDKDHC) is required for BCAA oxidation. BCKDHC consists of three catalytic components; the branched chain ketoacid dehydrogenase (BCKDH; alpha and beta subunit), dehydrolipoyl dehydrogenase (DLD) and dehydrolipoyl transacylase (DBT).

BCAA are transported via system L transporter containing a catalytic subunit and a glycoprotein 4F2hc/CD98. The subunit includes two isoforms called light chains LAT1 (gene: SLC7A5) and LAT2 (gene: SLC7A8) with high affinity to the BCAA [17,18].

Pathological high levels of BCAA have a critical influence on fetal growth and development in pregnant women suffering from maple syrup urine disease (MSUD) [19]. MSUD is an autosomal recessive disease with mutations in the gens Bckdha, Dld or Dbt. This genetic defect leads to a disturbed BCAA degradation. This disorder represents in neonates as a neurologic distress with either ketosis or ketoacidosis and hyperammonaemia.

For metabolic disorders like diabetes mellitus it is well known that the plasma amino acid concentrations are increased [20,21]. Hyperglycaemia in poorly controlled diabetes mellitus is related to high plasma levels of BCAA [22,23]. Likewise in pregnancies with gestational diabetes increases were observed for valine, methionine, phenylalanine, isoleucine, leucine, alanine in umbilical blood. So far, little is known about the effect of maternal diabetes on the embryonic amino acid content during early pregnancy.

We have used a rabbit animal model to investigate the effects of a type 1 diabetes mellitus on maternal and embryonic amino acid metabolism. We assumed that changes in the maternal plasma amino acid concentrations are transferred to the embryo prior to implantation as we had found high BCAA in early pregnant diabetic rabbits. Our study demonstrates that maternal diabetes mellitus type 1 leads to changes in amino acid composition and degradation in rabbits and their blastocysts during the preimplantation period with consequences on embryonic mTOR signalling.

## Materials and Methods

### Animals, embryo recovery and in vitro culture

#### Alloxan treatment

Experimental insulin dependent diabetes (IDD) was induced in female nonpregnant rabbits by alloxan treatment as described before [24]. The diabetic rabbits were hold in hyperglycaemic conditions with a permanent blood glucose concentration of >20 mmol/L for 9–11 days before mating. The blood glucose level was monitored twice per day. To prevent ketoacidosis insulin was supplemented 3 times per day (Humaninsulin basal, Lilly Deutschland GmbH, Giessen). This insulin supplementation is not enough to lower blood glucose levels to normal as diabetic rabbits should be investigated. For maternal blood and tissue analyses 6 or more pregnant rabbits from three different independent experiments were used. All blood samples were collected at day 6 after mating, at the same time when blastocysts and blastocyst cavity fluids were collected. In the same experiment and at the same time point (day 6.0 p.c.) blastocysts of three stages can be collected from individual donors (non-gastrulated stage 0 and early gastrulation stages 1 and 2).

All animal experiments were in accordance with the principles of laboratory animal care and had been approved by the local ethics committee (Landesverwaltungsamt Dessau, reference number 42502-2-812). All surgery was performed under Ketanest and Dormitor anesthesia and all efforts were made to minimize suffering.

#### Embryo recovery

Embryos were collected from sexually mature rabbits (12- to 16-weeks-old). The female rabbits, outbred ZIKA-hybrid New Zealand white, were purchased from a local breeder and were stimulated with 110 I.U. pregnant mare serum gonadotropin (PMSG, Intervet, Unterschleissheim, Germany) three days prior to mating. After mating 75 I.U. human choriongonadotropin (hCG, Intervet, Unterschleissheim, Germany) was intravenously injected to ensure ovulation. The rabbits were killed using an overdose of pentobarbital. Blood samples were collected with S-Monovetten (Sarstedt, Germany) containing EDTA. Plasma probes were centrifuged for 15 minutes at 4400 rpm and the supernatant were stored at -80°C until use. The uterine secretion was collected from the endometrial surface by using absorbing paper strips. The samples were stored at -80°C until use. Adipose tissue, skeletal muscle and liver were prepared and stored at -80°C until use for RNA and protein analyses.

#### Collection of blastocyst cavity fluids and sample generation for amino acid analyses

Collection of the cavity fluid was performed as followed: As rabbit blastocysts at day 6 are so big that they can be easily be identified by eye, they were picked up directly from the uterine luminal surface with a “blastocyst spoon”, briefly washed in cold PBS avoiding any storage and put on a 4°C precooled watch glasses. Blastocysts’ surface was then dried by flint-free cellulose paper. The blastocysts were staged and punctured and fluids were taken up with a pipette from the watch glasses and stored at -80°C until use. Overall, pick-up, rinsing and the collection of fluids and samples took less than 2 min and were highly standardized in all experimental replicates.

#### Embryo samples for RNA and protein analyses

The embryos were flushed from the oviduct or uteri on day 3, 4 and 6 *post coitum* (*p*.*c*.), washed two times in basal synthetic medium II (BSM II, serum- and growth factor-free) [25], pooled and randomly divided amongst the experimental groups. Six days old blastocysts were characterized morphologically and grouped by gastrulation stages in stage 0 (non-gastrulating), 1 (gastrulation stage 1 with anterior marginal crest) and 2 (gastrulation stage 2 with posterior gastrulation extension) [26]. Flushed embryos were washed with ice-cold phosphate buffered saline (PBS) and their gastrulation stage was determined. Embryos of gastrulation stage 1 and 2 were then transferred on a dry watch glass and washed again. After fluid removal the embryos were punctured and the effluent cavity fluid collected. Blastocyst cavity fluids were stored at -80°C until use.

For RNA isolation the embryos were washed 3 times in PBS containing 0.05% polyvinyl alcohol (PVA) and the extracellular coverings were removed mechanically. Samples of whole embryos or separated embryoblast and trophoblast were stored in PBS at -80°C until RNA isolation for RT-qPCR [27].

For Western Blot analysis embryos were washed 3 times in PBS and the extracellular coverings were removed mechanically in 0.05% PVA/PBS containing protease and phosphatase inhibitor. 8–10 embryos were pooled in RIPA buffer with protease and phosphatase inhibitor and stored at -80°C until further processing.

#### Embryo in vitro culture

To study the effects of insulin or glucose, groups of 3 to 6 blastocysts were cultured in 4 ml BSM II medium [25] at 37°C in a water saturated atmosphere of 5% O_2_, 5% CO_2_ and 90% N_2_ in a water-jacketed incubator (HERAcell 150i, Thermo Fisher Scientific, Bonn, Germany). After 2 hour preculture 100 μl culture medium with insulin for a final concentration of 17 nM (Life Technologies, Darmstadt, Germany) was added. The culture was continued for 1 or 4h. The experimental group embryos were handled equally but without insulin supplementation. The influence of glucose was analysed in blastocysts cultured with 0, 10 or 25 mM glucose in BSM II medium for 6 h. After culture the embryos were washed 3 times in ice cold PBS before the extracellular coverings were removed mechanically. Samples of whole embryos were stored as RNA sample at -80°C.

### Analysis of amino acids

The concentrations of free amino acids in maternal plasma, uterine secretion and in blastocyst cavity fluid were measured as isoindole derivatives by reversed phase high performance liquid chromatography (HPLC) (Hypersil ODS, 250 mm x 4 mm, 5 **μ**m, Agilent 1100, Agilent Technologies, Waldbronn, Germany) according to Schuster 1988 [28] with fluorescence detection (337 nm / 454 nm) after pre-column derivatisation with o-phthaldialdehyde and mercaptopropionic acid [29]. 20**μ**l of non-diluted plasma samples or 5**μ**l blastocyst cavity fluid (from single blastocysts) were precipitated with 0.25 vol sulphosalicylic acid (10%) and simultaneously incubated with 1 mM of internal standard nor-valine for 30 minutes at 4°C. Uterine secretions were eluated from the paper by adding of a defined volume of two parts deionized water and one part 1 mM internal standard nor-valine (final dilution 1:4) followed by an incubation in ice cold ultrasonic bath for 5 minutes. Afterwards the samples were centrifuged from the bored reaction tube into an imposed reaction tube for 5 minutes at 10000 g. Twenty **μ**l of the eluates were incubated with 5 **μ**l sulphosalicylic acid (10%) for 30 minutes at 4°C. All samples were centrifuged (18000 g, 10 min, 4°C) and the 1:5 (1:20 for uterine secretion samples) diluted supernatant was used for HPLC measurement.

### RNA isolation and cDNA synthesis

The mRNA of single blastocysts was extracted with Dynabeads Oligo (dT)_25_ (Life Technologies, Darmstadt, Germany) and used for cDNA synthesis [30]. The final volume of the cDNA reaction was adjusted with water to 100 μl for whole blastocysts and to 50 μl for separated embryoblast and trophoblast.

Total RNA from tissues (liver, skeletal muscle, adipose tissue and endometrium) was extracted by using TRIzol reagent (Life Technologies, Darmstadt, Germany) according to a previously described protocol [31]. Isolated RNA was treated with DNase for 30 minutes. The amount of total RNA was determined spectrophotometrically at 260 nm. Three μg of RNA was diluted in a total of 11.5 μl and preincubated with 1 μmol random pd(N)_6_ primer (Roche Diagnostics, Mannheim, Germany) at 65°C for 5 minutes. Afterwards the approach was reverse transcribed in a volume of 20 μl containing 200 units Revert Aid Reverse Transcriptase (Fermentas, St. Leon-Rot, Germany), 20 units RNase inhibitor, 1 mM dNTPs and 4 μl reaction buffer at 25°C 10 minutes, 42°C 60 minutes and 70°C 10 minutes.

### RT-qPCR

For real time analysis (RT-qPCR) duplicates of each cDNA sample and a no template control were measured for any primer set [32]. The primer sequences used in this study are documented in Table 1. The housekeeping gene glyceraldehyde 3-phosphate dehydrogenase (GAPDH) was quantified as endogenous control. Thieme *et al*. [27] had shown that GAPDH expression is not affected by the treatments used in current study. The target gene expression was normalized to that of GAPDH in each sample. A calibration curve was included from serial dilutions in a range from 10^8^–10^3^ copies of primer-specific DNA plasmid standards. Individual data are expressed relative to these standards.

**Table 1:** Oligonucleotides used for RT-PCR.

Gene	Primer sequence	Product [Bp]	Tm	Acc. No.
Rabbit GAPDH	fw: GCCGCTTCTTCTCGTGCAG rev: ATGGATCATTGATGGCGACAACAT	144	60°C	L23961
Rabbit Bcat2	fw: ACTACTCCCTGCAGCTCTTTG rev: GAACCCAGTCCTTGTCCACTC	191	60°C	XM_002723704.2
Rabbit Bckdha	fw: AGACAAGCTCGAGTTCATCCA rev: AGAACTTCAGCACCTTCTCCT	130	60°C	XM_002722294.1
Rabbit Dld	fw: GTGATAGGTTCTGGTCCTGGA rev: CAATTCCCCTAGATGCAAAAT	198	60°C	XR_085085.2
Rabbit Dbt	fw: GATATTGCCACCTGAAGTAGC rev: GAGAAGCGTGACCATTGAAGCA	155	60°C	XM_002715835.1
Rabbit SLC7A8	fw: GTCCCTTGCATCTGCTAAGACT rev: TCCAAAAAGTAAGGCATCCACT	234	60°C	NM_001082682.1
Rabbit SLC7A5	fw: CTCTTCCTCATCGCCGTCTC rev: TTTTTCCACCACACCCCGAA	110	60°C	NM_001082120.1

### Protein preparation and immunoblotting

Protein samples diluted in RIPA buffer were homogenized with Precellys (Peqlab, Erlangen, Germany), incubated 30 minutes on ice and afterwards centrifuged at 4°C for 20 minutes. The supernatant was used to measure the protein concentration according to Bradford. Western blot analyses were performed with 25 μg protein. After blotting, the nylon membranes were stained with Ponceau S. Molecular weights were determined by comparison with PageRuler prestained molecular weight marker (Fermentas, St. Leon-Rot, Germany). The immunoreactive signals were visualized by enhanced chemiluminescence detection (Millipore, Schwalbach, Germany) and quantified by Fusion Fx7 Imaging System (Peqlab, Erlangen, Germany). The primary antibody against BCAT2 (mouse polyclonal) was purchased from Abcam (ab72850) and diluted 1:1000. An anti-phospho-mTOR (Ser2448) (rabbit Ab, 1:500, #2971) and anti-mTOR (rabbit Ab, 1:500, #2972) antibody (both NEB, Frankfurt, Germany) were used to calculate phosphorylation as ratio of band intensities (p-mTOR vs. mTOR) in the same blot. Protein amount was evaluated by re-blotting with an antibody against the mouse monoclonal beta-actin antibody (Sigma, A-5441, 1:40000) after stripping the membranes. Protein amounts were calculated as ratio of band intensities (BCAT2 vs. beta-actin) in the same blot.

### Statistics

Data are expressed as mean value ± standard error of mean (mean ± SEM). If not stated otherwise, levels of significance between groups were calculated using student’s t-test after proving normal distribution. Multiple comparisons were made by factorial variance analysis (ANOVA) adjusted according to Bonferroni (statistical software: Sigma Plot v. 11.0). Levels of statistical significance are indicated as follows *- p<0.05, **- p< 0.01 and ***- p<0.001. All experiments were carried out at least three times.

## Results

### Amino acid concentrations in blood plasma of pregnant rabbits

The plasma amino acid composition from 11 diabetic rabbits and 11 non-diabetic healthy control females at day 6 of pregnancy was analysed and quantified by HPLC. In plasma samples from diabetic rabbits the concentrations of 12 amino acids were altered in comparison to controls. Alanine, valine, isoleucine, leucine and phenylalanine concentrations were significantly increased, whereas serine, glutamine, glycine, arginine, tyrosine, tryptophan and lysine concentrations were significantly reduced in diabetes (Table 2). Collectively, the concentrations of 5 essential amino acids were modified by maternal diabetes. The branched chain amino acids (BCAA) showed the most obvious effects. Their concentrations were approximately three-fold higher in plasma of diabetic rabbits than in plasma of the control group (Table 2).

**Table 2:** Concentration of free amino acids in blood plasma of diabetic (IDD) and non-diabetic (Control) rabbits at day 6 of pregnancy.

	Amino acid concentration [mM]			
Amino acid	Control	IDD	P	Fold change
**essential**				
Histidine	0.118 ± 0.003	0.116 ± 0.003		
Threonine	0.201 ± 0.011	0.248 ± 0.012		
Methionine	0.081 ± 0.004	0.089 ± 0.006		
Tryptophan	0.062 ± 0.002	0.055 ± 0.002	0.024	0.87
Phenylalanine	0.054 ± 0.001	0.061 ± 0.002	0.032	1.13
Lysine	0.203 ± 0.011	0.169 ± 0.007	0.02	0.83
Valine	0.241 ± 0.014	0.674 ± 0.095	0.001	2.8
Isoleucine	0.080 ± 0.005	0.231 ± 0.040	0.001	2.89
Leucine	0.108 ± 0.007	0.323 ± 0.057	0.001	2.99
**non-essential**				
Asparagine	0.106 ± 0.005	0.091 ± 0.008		
Serine	0.247 ± 0.006	0.209 ± 0.012	0.014	0.85
Glutamine	0.836 ± 0.023	0.527 ± 0.017	0.001	0.63
Glycine	0.903 ± 0.041	0.500 ± 0.042	0.001	0.55
Arginine	0.211 ± 0.016	0.112 ± 0.014	0.001	0.53
Alanine	0.355 ± 0.028	0.624 ± 0.065	0.01	1.75
Taurine	0.128 ± 0.023	0.092 ± 0.009		
Tyrosine	0.079 ± 0.002	0.053 ± 0.002	0.001	0.68
Ornithine	0.063 ± 0.003	0.085 ± 0.013		

Results are given in mM and are from 3 independent experiments including 11 individuals in each group. (mean ± SEM; N = 3; n = 11).

### Amino acid concentrations in uterine secretion of pregnant rabbits

Amino acid composition of uterine secretion from 7 diabetic rabbits and 8 non-diabetic healthy control females at day 6 of pregnancy was analysed and quantified by HPLC. The concentrations of threonine, valine, isoleucine, leucine, glutamine, arginine, alanine and taurine were significantly increased in uterine secretion of diabetic rabbits (Table 3).

**Table 3:** Comparison of mean values of amino acids measured in uterine secretions from diabetic (IDD) and non-diabetic (Control) rabbits.

	Amino acid concentration [mM]			
Amino acid	Control	IDD	P	Fold change
**essential**				
Threonine	0.651 ± 0.050	0.803 ± 0.043	0.043	1.23
Methionine	0.205 ± 0.026	0.213 ± 0.018		
Tryptophan	0.048 ± 0.003	0.050 ± 0.005		
Phenylalanine	0.151 ± 0.020	0.164 ± 0.015		
Lysine	0.355 ± 0.034	0.342 ± 0.029		
Valine	0.350 ± 0.033	0.573 ± 0.062	0.002	1.63
Isoleucine	0.157 ± 0.019	0.238 ± 0.029	0.032	1.51
Leucine	0.269 ± 0.023	0.454 ± 0.055	0.017	1.69
**non-essential**				
Serine	1.994 ± 0.148	2.032 ± 0.141		
Glutamine	0.046 ± 0.008	0.100 ± 0.022	0.04	2.15
Glycine	34.964 ± 3.136	41.073 ± 1.660		
Arginine	0.181± 0.025	0.262 ± 0.027	0.021	1.44
Alanine	4.307 ± 0.413	6.884 ± 0.267	0.001	1.60
Taurine	0.733 ± 0.066	1.008 ± 0.098	0.033	1.37
Tyrosine	0.176 ± 0.018	0.148 ± 0.015		
Ornithine	0.076 ± 0.016	0.054 ± 0.005		

Results are given in mM and are from 4 independent experiments. (mean ± SEM; N = 4; n_control_ = 8; n_IDD_ = 7). and non-diabetic (p< 0.05); b—significantly different in gastrulation stage 1 and 2 (p< 0.05)).

### Amino acid concentrations in the blastocyst cavity fluid of 6 days old blastocysts at gastrulation stage 1 and 2

Blastocyst cavity fluid (BCF) was collected from embryos at gastrulation stage 1 and 2. BCF of 11 individual blastocysts from diabetic and non-diabetic females was analysed and quantified in each group and stage by HPLC. The amino acid concentrations (Mean±SEM) are listed in Table 4.

**Table 4:** Comparison of mean values of amino acids measured in blastocyst cavity fluid from diabetic (IDD) and non-diabetic (Control) rabbits at gastrulation stages 1 and 2.

Amino acid concentration [mM]				
	Stage 1		Stage 2	
Amino acid	Control	IDD	Control	IDD
**essential**				
Histidine	0.094 ± 0.013	0.080 ± 0.007	0.094 ± 0.007	0.112 ± 0.009 **b**
Threonine	0.337 ± 0.025	0.302 ± 0.028	0.387 ± 0.022	0.453 ± 0.021 **b**
Methionine	0.113 ± 0.020	0.114 ± 0.023	0.143 ± 0.023	0.116 ± 0.015
Tryptophan	0.050 ± 0.004	0.040 ± 0.03	0.055 ± 0.005	0.048 ± 0.006
Phenylalanine	0.066 ± 0.005	0.054 ± 0.003	0.073 ± 0.006	0.067 ± 0.005
Lysine	0.438 ± 0.044	0.483 ± 0.027	0.411 ± 0.050	0.582 ± 0.061 **a**
Valine	0.268 ± 0.030	0.586 ± 0.054 **a**	0.271 ± 0.026	0.595 ± 0.052 **a**
Isoleucine	0.084 ± 0.010	0.265 ± 0.029 **a**	0.099 ± 0.011	0.240 ± 0.024 **a**
Leucine	0.112 ± 0.011	0.373 ± 0.043 **a**	0.133 ± 0.013	0.330 ± 0.033 **a**
**non-essential**				
Asparagine	0.341 ± 0.033	0.250 ± 0.019	0.286 ± 0.024	0.367 ± 0.029 **b**
Serine	0.892 ± 0.108	0.720 ± 0.039	0.992 ± 0.076	1.098 ± 0.069 **b**
Glutamine	0.491 ± 0.065	0.798 ± 0.084 **a**	0.522 ± 0.042	0.754 ± 0.062 **a**
Glycine	5.414 ± 0.352	7.688 ± 0.767 **a**	6.451 ± 0.408	7.770 ± 0.029 **a**
Arginine	0.318 ± 0.050	0.316 ± 0.018	0.248 ± 0.016	0.380 ± 0.038 **a**
Alanine	2.333 ± 0.311	3.949 ± 0.508 **a**	2.063 ± 0.234	3.851 ± 0.406 **a**
Taurine	0.093 ± 0.007	0.161 ± 0.015 **a**	0.086 ± 0.005	0.141 ± 0.025 **a**
Tyrosine	0.090 ± 0.007	0.047 ± 0.003 **a**	0.092 ± 0.007	0.061 ± 0.004 **ab**
Ornithine	0.089 ± 0.019	0.093 ± 0.007	0.193 ± 0.027 **b**	0.154 ± 0.015 **b**

Results are given in mM and are from 3 independent experiments including 11 individuals in each group. (mean ± SEM; ANOVA; N = 3; n = 11; a—significantly different between diabetic and non-diabetic (p< 0.05); b — significantly different in gastrulation stage 1 and 2 (p< 0.05)).

We compared the amino acid composition in blastocyst cavity fluid at gastrulation stage 1 and stage 2. In blastocysts from healthy rabbits only the concentration of the non-essential amino acid ornithine was different with a two-fold increase in stage 2 (Table 4). In blastocysts from diabetic rabbits we found an increase of asparagine, serine, histidine, threonine, tyrosine and ornithine at gastrulation stage 2 (Table 4).

The concentrations of 8 amino acids (3 essential and 5 non-essential amino acids) were altered in the blastocyst cavity fluid from gastrulation stage 1 blastocysts grown in diabetic mothers compared to control blastocysts of the same stage. Concentrations of 7 amino acids (glutamine, glycine, alanine, taurine, valine, isoleucine and leucine) were significantly higher. Tyrosine concentration was decreased. Remarkably, as in maternal plasma, the BCAA showed the strongest differences in BCF of embryos from diabetic rabbits in comparison to the controls. Embryonic BCAA were two- to three-fold increased, while other essential amino acids were not changed. In blastocysts at gastrulation stage 2 the concentrations of 10 amino acids (4 essential and 6 non-essential amino acids) were altered under diabetic conditions (Table 4). Tyrosine was reduced. BCAA and lysine were increased. The non-essential amino acids glutamine, glycine, arginine, alanine and taurine were significantly enhanced in blastocysts of diabetic rabbits. Again, the strongest effect was found in BCAA.

### Expression pattern of BCAA oxidizing enzymes in maternal tissue

Diabetes showed a strong impact on the BCAA concentrations. We assumed that the increase in BCAA concentrations was caused by a disruption of BCAA degradation due to transcriptional regulation in maternal tissues as described by Herman in mice before [33]. Consequently, the embryo should adapt its amino acid metabolism to the increased BCAA availability.

The mRNA amounts of the BCAA oxidizing enzymes branched chain aminotransferase 2 (Bcat2), 2-oxoisovalerate dehydrogenase subunit alpha (Bckdha), dehydrolipoyl dehydrogenase (Dld) and dehydrolipoyl transacylase (Dbt) were analysed in various maternal tissues (liver, skeletal muscle and adipose tissue). The lowest expression level of all enzymes was found in the skeletal muscle (Bcat2: 13±1, Bckdha: 2±0.09, Dld: 408±41, Dbt: 26±3 molecules per 10^5^ GAPDH molecules) and the highest in liver (Bcat2: 104±11, Bckdha: 123±10, Dld: 38090±2800, Dbt: 14757±1225 molecules per 10^5^ GAPDH molecules) and adipose tissue (Bcat2: 275±25, Bckdha: 1666±88, Dld: 19041±1488, Dbt: 3839±172 molecules per 10^5^ GAPDH molecules). Bcat2 was higher expressed in adipose tissue than in liver.

**Fig 1:**
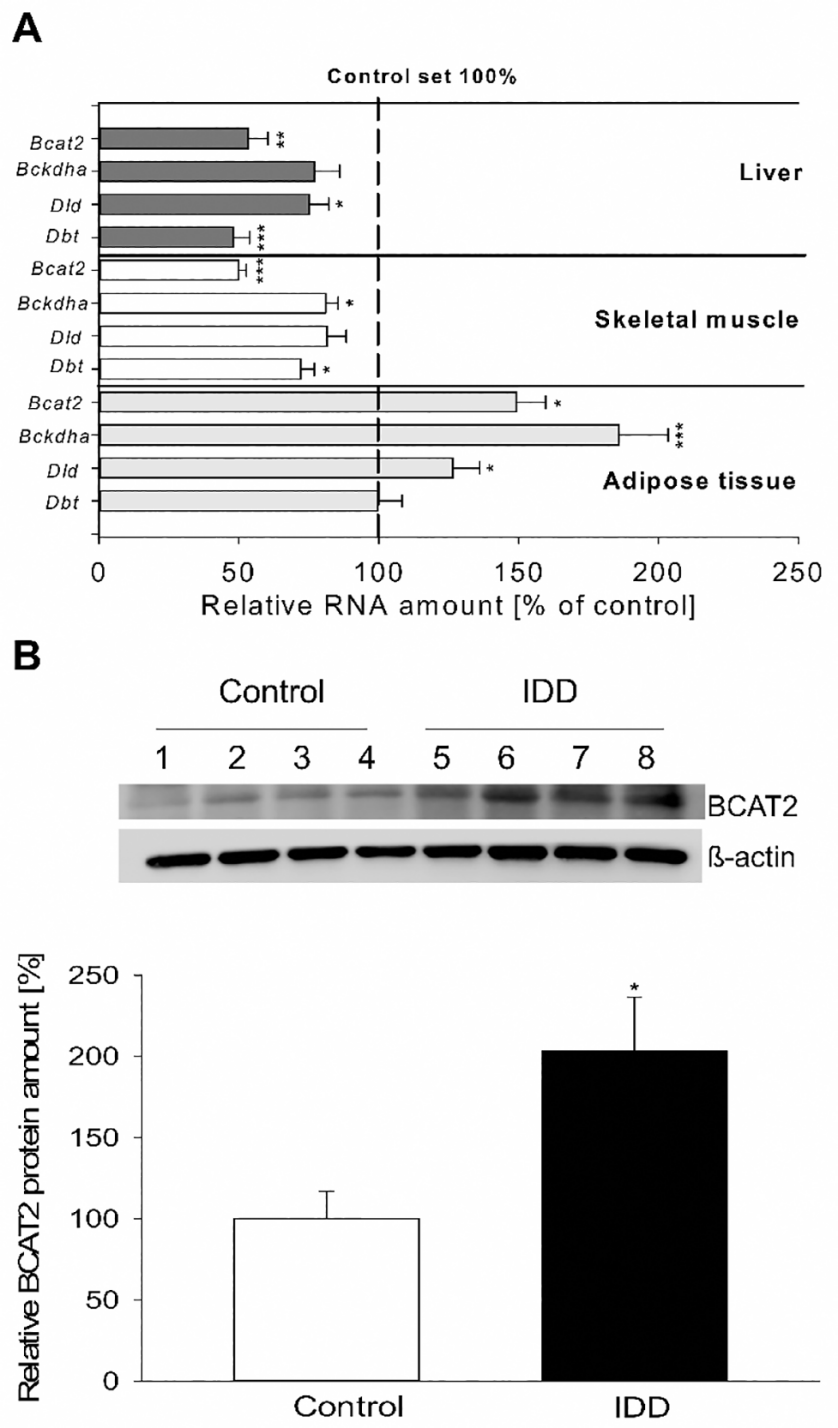
Expression of BCAA oxidizing enzymes in tissues of diabetic (IDD) rabbits. (A) Transcript amounts of Bcat2, Bckdha, Dld and Dbt were measured in liver, skeletal muscle and adipose tissue from diabetic and non-diabetic (control) rabbits at day 6 *p*.*c*. by real time RT-qPCR. The control group is set 100%. Transcript amounts of most oxidizing enzymes were reduced in liver and skeletal muscle and enhanced in adipose tissue of diabetic rabbits in comparison to healthy controls. (B) BCAT2 protein amount was analysed by Western blotting in adipose tissue of diabetic (IDD) and non-diabetic (control) rabbits. A representative Western blot for BCAT2 is shown. Amounts were calculated relative to ß-actin of the same sample. BCAT2 amounts of the control group are set 100%. Diabetic rabbits showed an approximately 2 fold higher BCAT2 protein amount in adipose tissue than healthy controls. (1,2,3,4—individual protein samples isolated from non-diabetic rabbits; 5,6,7,8—individual protein samples isolated from diabetic rabbits; N = 3; n ≥ 6; * = p˂ 0.05; ** = p< 0.01; ***p = <0.001).

In diabetic rabbits the Bcat2 mRNA level was increased in adipose tissue compared to the controls (Fig 1A). In adipose tissue mRNA amounts of the Bckdha and the Dld were increased, too. However, the expression of most oxidizing enzymes was reduced in liver and skeletal muscle. The analysis of BCAT2 protein amount revealed a two-fold increase in adipose tissue of diabetic rabbits compared to controls (Fig 1B and 1C).

### Expression of BCAA oxidizing enzymes and amino acid transporter in preimplantation embryos

**Fig 2:**
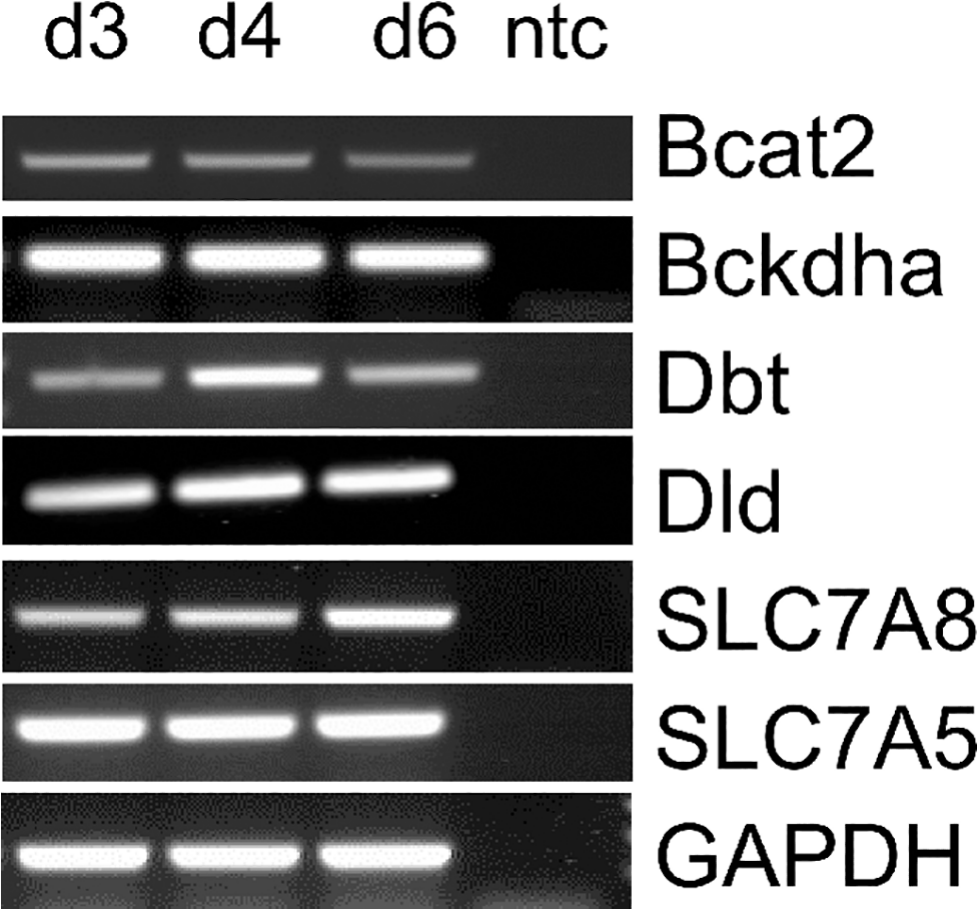
Expression of BCAA oxidizing enzymes and amino acid transporters in rabbit embryos. Expression of Bcat2, Bckdha, Dld, Dbt, SLC7A8 and SLC7A5 was determined in preimplantation rabbit embryos at day 3 (d3; morula), 4 (d4; early blastocyst) and 6 *p*.*c*. (d6; expanded blastocyst) by RT-qPCR. Transcripts of BCAA oxidizing enzymes and transporters were present in all investigated stages. (ntc—non template control).

**Fig 3:**
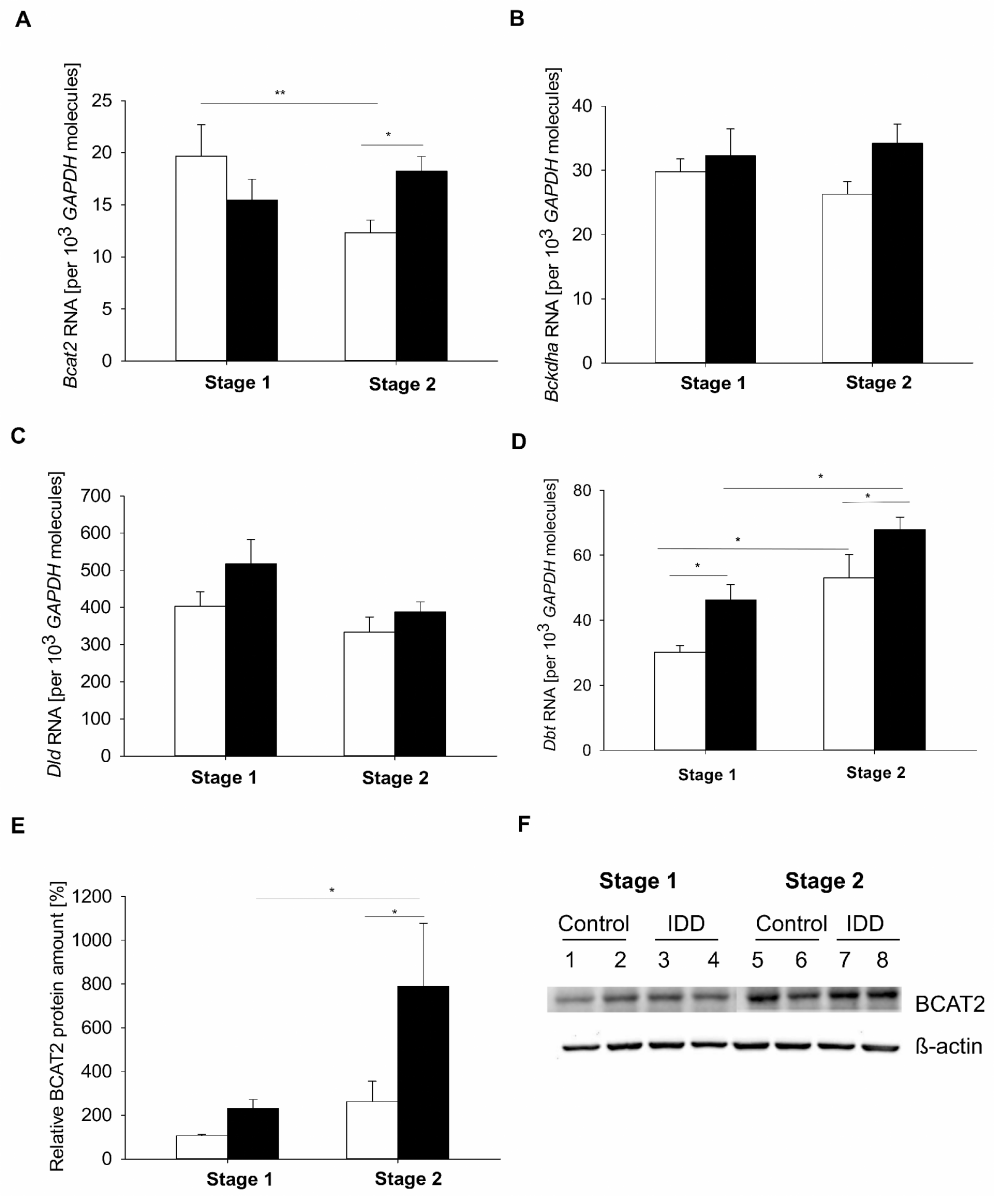
Expression of BCAA oxidizing enzymes in blastocysts of diabetic (IDD) rabbits. The mRNA amounts of Bcat2, Bckdha, Dld and Dbt were quantified by real time RT-qPCR in blastocysts of gastrulation stage 1 and 2 from control (white bars) and diabetic rabbits (black bars). Bcat2 and Dbt transcripts were significantly increased in stage 1 and stage 2 embryos from diabetic rabbits (A+D) compared to control blastocysts; Dld and Bckdha transcripts were not influenced by maternal diabetes (B+C). (ANOVA; N = 3; n≥ 9; * = p˂ 0.05). (E) The relative BCAT2 protein amount was analysed by Western blotting in blastocysts of diabetic (IDD; black bars) and non-diabetic (control; white bars) rabbits. ß-actin was used as loading control. The control group stage 1 is set 100%. Blastocysts of diabetic rabbits revealed an approximately two-fold increase in BCAT2 protein amount in both gastrulation stages. (F) A representative Western Blot for BCAT2 is shown with protein samples from 10 pooled blastocysts per group (1–8). (ANOVA; N = 4; n = 40; * = p< 0.05).

Fig 2 documents the ontogenetic pattern of BCAA transporter system L with the subunits LAT1 (gene: SLC7A5) and LAT2 (gene: SLC7A8) in morulae (day 3), early blastocyst (day 4) and expanded blastocyst (day 6). Transcripts of SLC7A5 and SLC7A8 and the BCAA oxidizing enzymes Bcat2, Dld, Dbt and Bckdha were present in all investigated embryo stages (Fig 2). Bcat2 mRNA amount was quantified by real time RT-PCR in separated embryoblast (16.03±1.43 molecules per 10^3^ *GAPDH* molecules) and trophoblast (15.31±1.63 molecules per 10^3^ *GAPDH* molecules). Bcat2 transcripts were equal in both cell lineages. During gastrulation at day 6, blastocysts Bcat2 transcript levels decreased from stage 1 to 2, whereas Dbt transcripts increased (Fig 3A and 3D). The mRNA amount of Bckdha and Dld was not affected (Fig 3B and 3C).

### Expression of BCAA oxidizing enzymes in preimplantation embryos from diabetic rabbits

In blastocysts grown in diabetic rabbits the transcript and protein amounts of BCAT2 were significantly increased compared to control blastocysts (Fig 3). Also Dbt-RNA amount was significantly enhanced in gastrulation stages 1 and 2 compared to corresponding control blastocysts (Fig 3D). The transcription of Bckdha and Dld was not influenced by maternal diabetes (Fig 3B and 3C).

### Expression of Bcat2 in in vitro cultured preimplantation embryos

**Fig 4:**
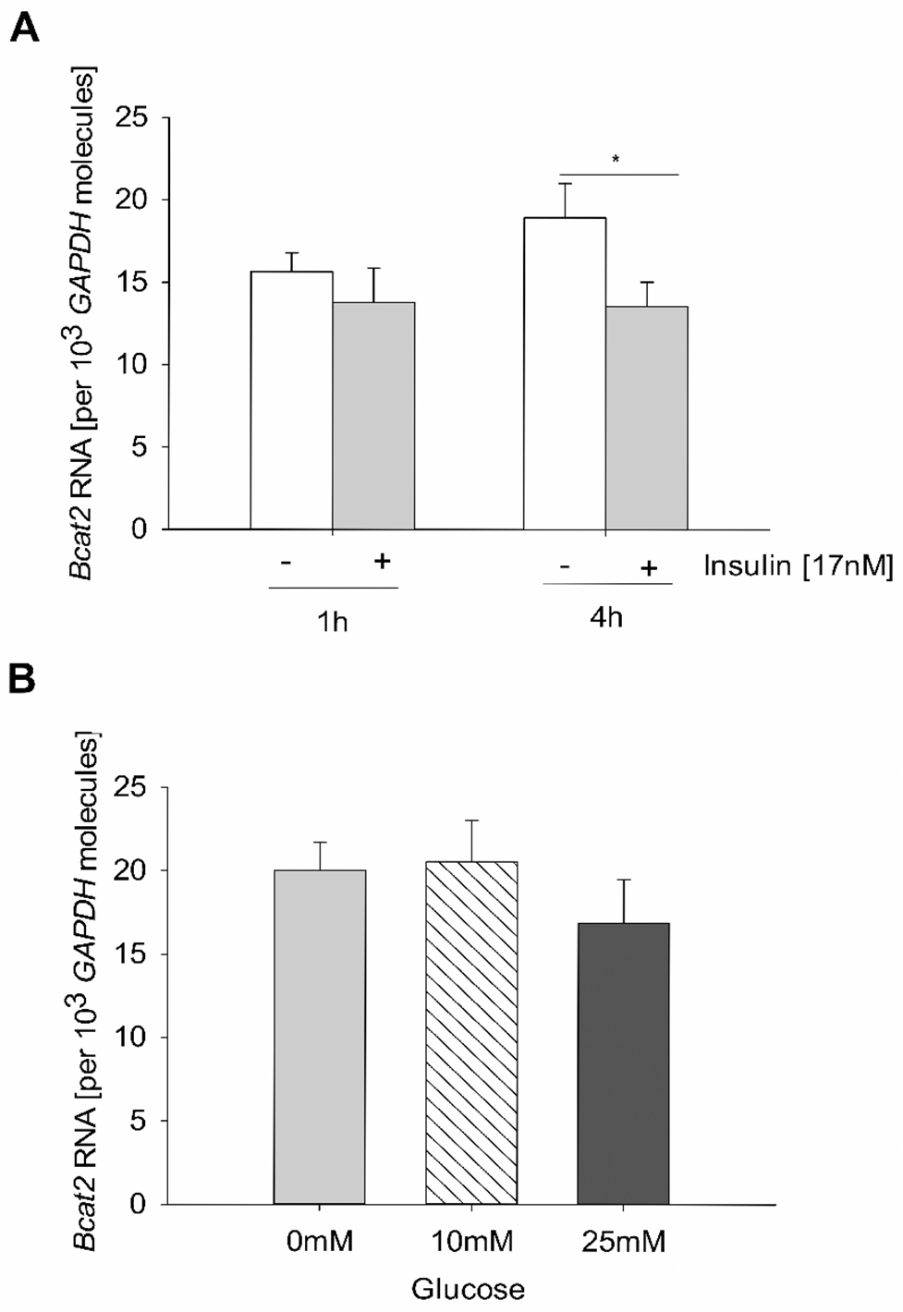
Expression of Bcat2 in blastocysts cultured in vitro without insulin and with different glucose concentrations. Blastocysts were cultured in pools of 3 to 6 embryos with 17 nM insulin (A) or in 0, 10 or 25 mM glucose. The mRNA amount of Bcat2 was quantified by real time RT-qPCR in single blastocysts. The Bcat2 transcript level was unaltered after 1 h culture without or with insulin and increased after 4 h culture in the insulin free group (A). Bcat2 transcript amounts were not altered after culture with glucose for 6h (B). (N = 3; n≥ 11; * = p< 0.05).

The quantification of Bcat2 RNA in blastocysts cultured without insulin showed higher amounts after 4 h in vitro culture (Fig 4A). A 6 h culture with 0, 10 or 25 mM glucose had no effect on embryonic Bcat2 expression.

### Expression of amino acid transporters in endometrium and preimplantation embryos from diabetic rabbits

**Fig 5:**
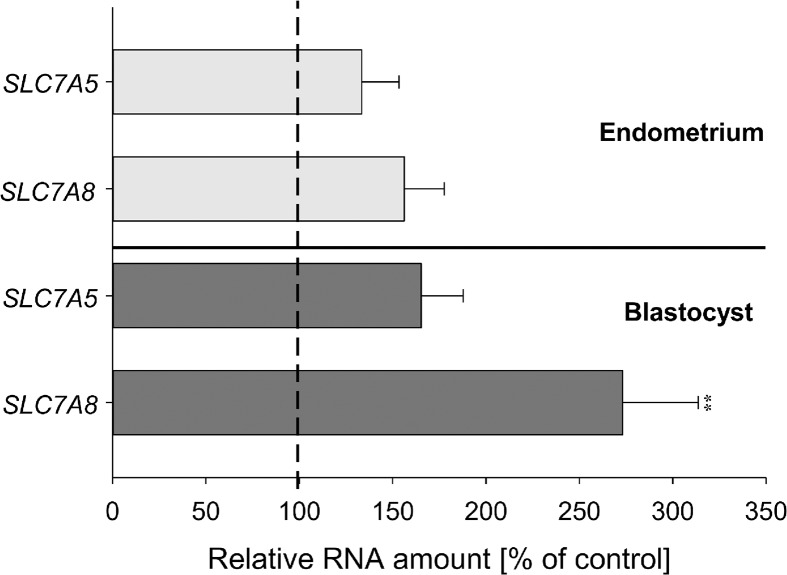
Expression of amino acid transporters in endometrium and blastocysts of diabetic rabbits. Transcript amounts of SLC7A5 and SLC7A8 were measured in endometrium and blastocysts of the gastrulation stage 2 from diabetic and non-diabetic (control) rabbits at day 6 p.c. by real time RT-qPCR. The control group was set 100%. In blastocysts developed under diabetic conditions the SLC7A8 transcript amount was significantly increased but not SLC7A5 and none in endometrium. (N = 3; n≥ 9; ** = p< 0.005).

SLC7A5 and SLC7A8 mRNA amounts were quantified by real time RT-qPCR in endometrium of diabetic rabbits and their blastocysts at gastrulation stage 2. SLC7A5 and SLC7A8 transcript amounts were not statistically significantly different in endometrium of control and diabetic rabbits (Fig 5). In blastocysts grown in diabetic rabbits the transcript amount of SLC7A5 was unaltered, whereas SLC7A8 transcripts were significantly increased in blastocysts from diabetic rabbits compared to blastocysts of healthy controls (Fig 5).

### mTOR in preimplantation embryos developed under diabetic conditions

**Fig 6:**
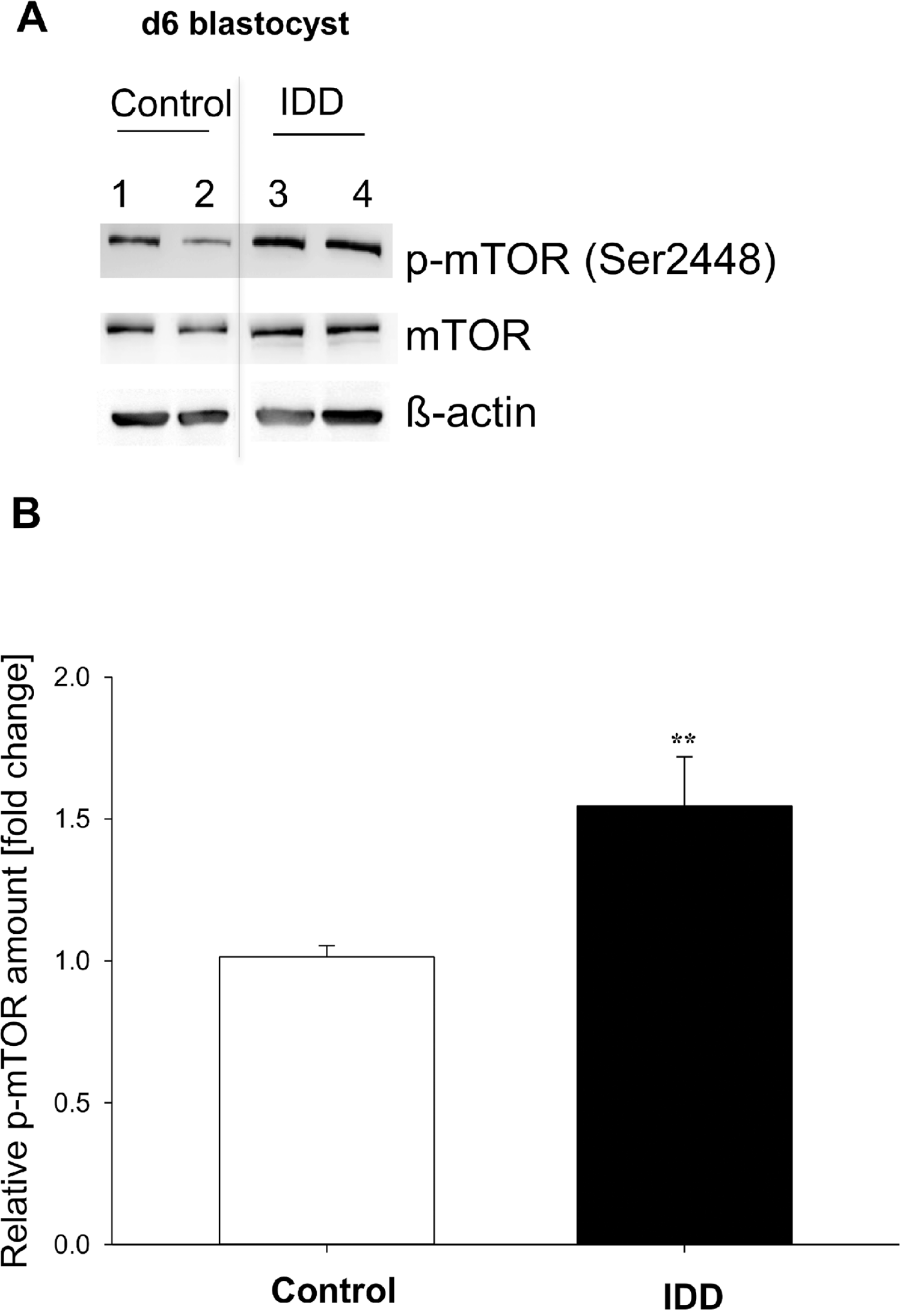
Phosphorylation of mTOR in blastocysts of diabetic (IDD) rabbits. The relative amount of mTOR phosphorylation was quantified by Western blotting in four samples pooled from a total of 30 blastocysts of gastrulation stage 2. (A) shows a representative Western Blot of two pooled samples (n≥ 7 blastocysts per sample) from blastocysts from control (lanes 1,2) and diabetic (lanes 3,4) rabbits. The amount of phosphorylated protein was related to the non-phosphorylated protein and calculated relative to the healthy blastocysts (set 1). The phosphorylation of mTOR was significantly increased in stage 2 blastocysts of diabetic rabbits compared to controls (B). (N = 4; n = 30; ** = p< 0.005).

Blastocysts from diabetic rabbits revealed an approximately 1.5-fold higher phosphorylation of mTOR compared to healthy controls (Fig 6). The protein amount of mTOR was not influenced by maternal diabetes.

## Discussion

In diabetic patients with poorly controlled hyperglycaemia the plasma levels of amino acids are altered. The plasma BCAA levels are increased and serine and glycine concentrations are reduced by a diabetic ketoacidosis [34]. A comparable change in amino acid pattern was also observed in nonobese patients with insulin-dependent diabetes mellitus [35]. The plasma amino acid concentrations are comparable between humans and rabbits. In human plasma samples the normal concentrations for leucine, isoleucine and valine are 0.14±0.03 mM, 0.07±0.01 mM and 0.25±0.002 mM, respectively [22]. In diabetic ketosis the concentrations of BCAA are two-fold increased (0.27–0.28 mM leucine, 0.14–0.15 mM isoleucine, 0.40–0.46 mM valine) [22,34]. In current study, rabbits with an induced diabetes mellitus type 1 showed the same pattern of plasma amino acids as diabetic humans (Table 2). The two-fold increase in BCAA in blood as a consequence of diabetes has also been described in two experimental diabetic rat models, the Zucker diabetic fatty rat [36] and in streptozotocin induced diabetes [37].

So far, little is known about amino acid metabolism of mammalian embryos during the pre-implantation period. We used rabbit gastrulating blastocysts at stage 1 and 2 for our study.

In blastocysts from healthy donors the amino acid composition was comparable between both developmental stages, indicating the stability of embryonic amino acid metabolism at those stages. Only ornithine was found in a higher concentration in the cavity fluid of stage 2 blastocysts. Ornithine is required for polyamine synthesis, which is reported to be essential for embryonic growth [38]. In rabbit blastocysts the concentrations of the gluconeogenic amino acids alanine and glycine were six- to eight-fold higher compared with maternal plasma concentrations. Beside asparagine and glutamic acid the concentrations of alanine and glycine were also the highest in bovine blastocoel fluid compared to control synthetic oviductal fluid [39]. Early embryos are characterised by elevated levels of both amino acids. In hamster embryos it has been hypothesized that glycine and alanine are important for intracellular pH regulation by acting as proton shuttles [40]. The rabbit blastocyst is one of the biggest mammalian blastocysts with a strong need for alanine and glycine for expansion.

Current study shows for the first time the influence of maternal diabetes on embryonic amino acid metabolism prior to implantation. In contrast to normal development conditions stage 2 blastocysts grown in a diabetic milieu have an altered amino acid pattern with increased concentrations of BCAA, arginine, alanine, lysine, glutamine, glycine and taurine in blastocoel fluid, indicating that the embryonic amino acid composition is closely related to maternal amino acid plasma levels and uterine supply.

Further, diabetic conditions during gastrulation are characterised by increased ornithine, asparagine, serine, histidine, threonine and tyrosine concentrations in rabbit embryos. The reasons remain unclear. Some of these amino acids are involved in cellular stress protection, indicating that diabetic conditions act as a metabolic stress during this ontogenetic stage.

**Fig 7:**
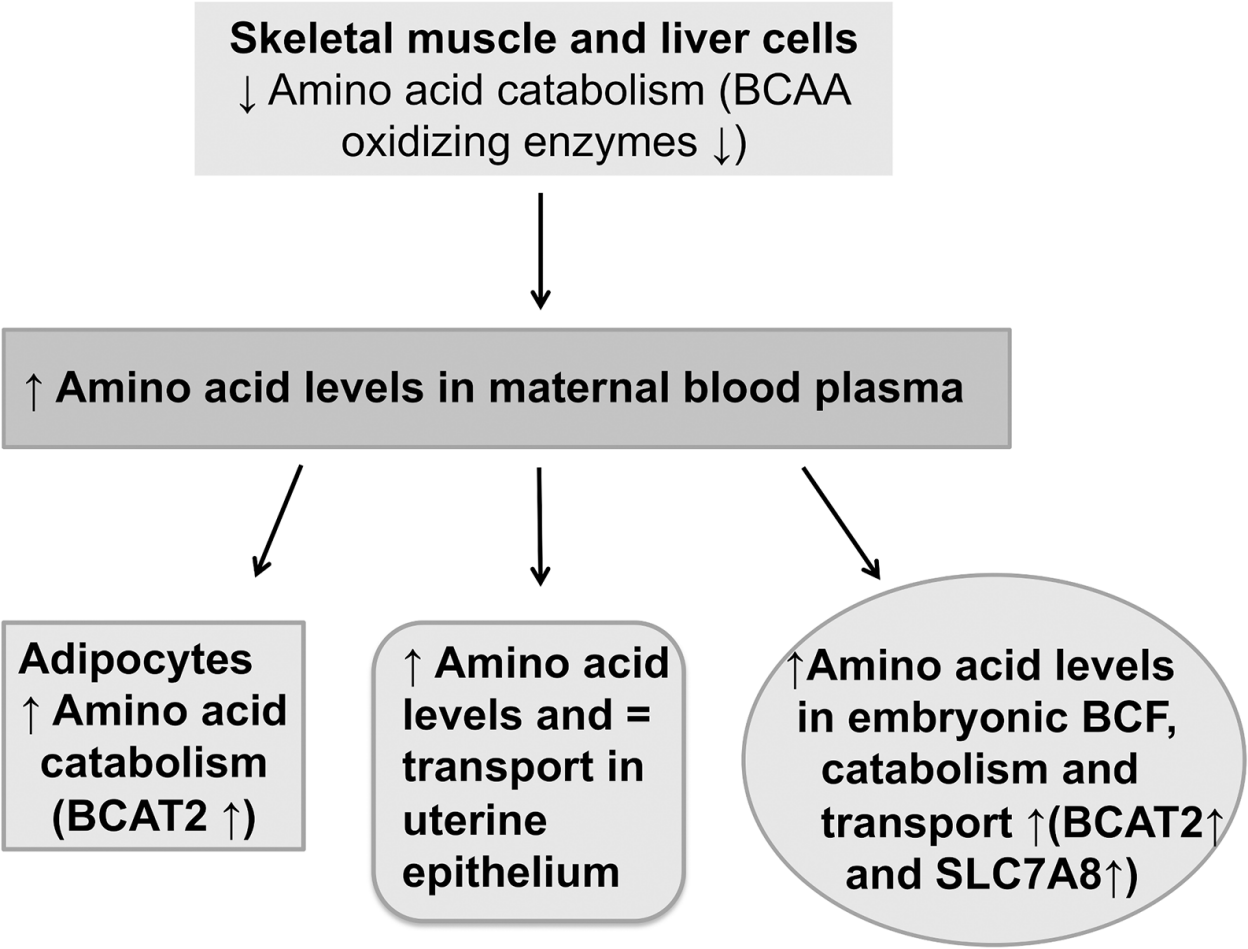
Schematic model of amino acid catabolism in maternal tissues and embryos in a diabetic pregnancy. Maternal diabetes leads to reduced BCAA catabolism in skeletal muscle and liver and to increased BCAA catabolism in adipose tissue. As a result of the reduced catabolism in muscle tissue the maternal blood plasma BCAA levels are enhanced. This might influence the BCAA levels in uterine secretions as BCAA are transferred via SLC7A8 to the uterine lumen and to embryos. BCAA accumulate in the blastocyst cavity fluid (BCF) and can be used as a source for embryonic metabolism and protein synthesis, increasing the embryonic BCAA supply.

We hypothesise a disturbed BCAA degradation in skeletal muscle and liver cells as potential reason for BCAA accumulation in maternal plasma, which may lead to an increased catabolism in maternal adipocytes. A scheme of BCAA catabolism in diabetic pregnancy is given in Fig 7. Our analysis revealed a reduced transcription level of all investigated BCAA oxidizing enzymes (Bcat2, Bckdha, Dbt and Dld) in liver and skeletal muscle from diabetic rabbits. The reduced degradation of BCAA in skeletal muscle, which accomplishes 60% of the BCAA oxidizing capacity [13], and in the liver leads to a BCAA accumulation in plasma during diabetes (Fig 7). Conversely, the transcript and protein amounts of aminotransferase BCAT2, which catalyses the first step of BCAA degradation, were elevated in adipose tissue. The adipose tissue may benefit from high BCAA. The increase in BCAA oxidizing enzymes may enable adipose tissue to produce more lipids [41].

In embryos from diabetic rabbits the changes in expression of BCAA oxidizing enzymes were quantified. These embryos showed higher mRNA amounts of Bcat2 like in maternal adipose tissue. Both, transcripts and protein of BCAT2 were increased, indicating a higher embryonic transamination of BCAA to their corresponding keto acid. Also Dbt expression was enhanced in embryos from diabetic rabbits, while the enzymes of the BCKDH complex were transcriptional unaltered. In conclusion, these data demonstrate a higher embryonic amino acid catabolism in a diabetic pregnancy (Fig 7).

It is known that BCAA are sensitive to changes in insulin [41]. For example, in human postoperative patients, diabetic or obese, supplementation of insulin returns plasma BCAA to normal levels [42,43,44]. The insulin supplementation in our rabbit model, securing survival under diabetic metabolic conditions, is not high enough to reduce BCAA levels to the normal. In obese mice and human co-twins with normal or high insulin levels the BCAT2 expression was decreased underlying that BCAT2 is regulated by insulin [33,45]. In adipose tissue of diabetic rabbits we measured an increased Bcat2 transcription. This increase was closely correlated with increased plasma BCAA levels. In contrast, Bcat2 transcripts were lower in skeletal muscle and liver tissues, suggesting a tissue specific Bcat2 regulation. As shown by in vitro culture experiments insulin availability regulated Bcat2 transcription in preimplantation embryos, whereas glucose did not. Insulin supplementation in vitro kept the embryonic Bcat2 transcription down, while hypoinsulinaemic conditions in vivo increased Bcat2 transcription in blastocysts from diabetic mothers. We suggest that insulin deficiency is a potential and likely reason for higher Bcat2 transcription in embryos from diabetic mothers.

Changes in maternal metabolism may affect the early embryo by various ways, with uterine epithelia playing key role. Analysis of nutrients in the uterine fluid revealed the presents of amino acids and an increase of amino acids in uterine fluid compared to plasma levels in mice [46,47]. Higher BCAA levels were found in females after fertilisation [48]. In our study the concentrations of BCAA were increased in uterine secretions of diabetic rabbits, which was reflected by higher BCAA levels in their embryos, too. However, we observed no alterations in endometrial BCAA transporters while in blastocysts of diabetic donors, the LAT2 transporter was transcriptional enhanced. This indicates a higher embryonic BCAA transport, which may have contributed to the higher embryonic BCAA levels.

Maternal metabolic conditions are transferred to the developing embryo as early as during the preimplantation period. Altered patterns of BCAA concentrations as a consequence of metabolic disorders have a potential influence on embryonic development programming mechanisms. The accumulation of leucine or its metabolites appears to be the main cause of neurotoxicity like in MSUD and may result in neurological dysfunction in the long term [49,50]. It has been suggested that the viability of mammalian embryos is associated with a metabolism that is quiet rather than active during early development [51]. In human early embryos it is known that a reduced amino acid turnover is important for the blastocyst formation in vitro [52]. Diabetic conditions as shown in current study resulted in an increase in amino acid catabolism, disturbing the “quietness” of the embryo and its developmental competence or/and metabolic imprinting as embryos that are metabolically too active may be of poorer quality/viability.

During maternal protein restriction reduced plasma BCAA levels resulted in intrauterine growth restriction (IUGR) and affected the metabolic health of adult offspring [53]. BCAA supplementation reverses IUGR impact during pregnancy [54,55]. Mice fed with a low protein diet and their blastocysts at day 3.5 have decreased BCAA levels [56]. BCAA are necessary for various metabolic activities (energy production as pyruvate, protein synthesis and degradation [57,58]. Especially leucine is a potent activator of the mammalian target of rapamycin (mTOR). We evaluated directly the effect of maternal diabetes on embryonic mTORC1 signalling using antibodies to detect relative pools of phosphorylated and total mTOR. Central finding of this study is the increased activation of mTOR in blastocysts developed under diabetic conditions. Potential effects of altered mTOR signalling on protein synthesis and trophoblast outgrowth have been proposed [59,60]. An altered mTOR signalling based on the high BCAA levels in rabbit preimplantation embryos might be an important mechanism with long-lasting programming effects on pre- and postnatal development.
